# Sequestration of synaptic proteins by alpha-synuclein aggregates leading to neurotoxicity is inhibited by small peptide

**DOI:** 10.1371/journal.pone.0195339

**Published:** 2018-04-02

**Authors:** Mal-Gi Choi, Mi Jin Kim, Do-Geun Kim, Ri Yu, You-Na Jang, Won-Jong Oh

**Affiliations:** Department of Structure and Function of Neural Network, Korea Brain Research Institute, Daegu, South Korea; Louisiana State University Health Sciences Center, UNITED STATES

## Abstract

α-Synuclein (α-syn) is a major component of Lewy bodies found in synucleinopathies including Parkinson’s disease (PD) and Dementia with Lewy Bodies (DLB). Under the pathological conditions, α-syn tends to generate a diverse form of aggregates showing toxicity to neuronal cells and able to transmit across cells. However, mechanisms by which α-syn aggregates affect cytotoxicity in neurons have not been fully elucidated. Here we report that α-syn aggregates preferentially sequester specific synaptic proteins such as vesicle-associated membrane protein 2 (VAMP2) and synaptosomal-associated protein 25 (SNAP25) through direct binding which is resistant to SDS. The sequestration effect of α-syn aggregates was shown in a cell-free system, cultured primary neurons, and PD mouse model. Furthermore, we identified a specific blocking peptide derived from VAMP2 which partially inhibited the sequestration by α-syn aggregates and contributed to reduced neurotoxicity. These results provide a mechanism of neurotoxicity mediated by α-syn aggregates and suggest that the blocking peptide interfering with the pathological role of α-syn aggregates could be useful for designing a potential therapeutic drug for the treatment of PD.

## Introduction

Protein aggregation is a hallmark in age-associated neurodegenerative diseases such as Parkinson’s disease (PD), Alzheimer’s disease (AD), Huntington’s disease (HD), and amyotrophic lateral sclerosis (ALS) [1]. Generally, a number of severe pathological conditions, genetics, and environmental factors are involved in abnormal protein aggregation [2]. Aggregating proteins (*i*.*e*. α-syn, Aβ, and tau) normally exist in the brain as monomers, however, typically form amyloid fibrils with age in the neurodegenerative diseases patient brain [3]. In the process of fibrillar amyloid aggregation, soluble oligomeric forms of intermediates have been considered as a neurotoxic form [3]. Besides aging, mutations in these proteins and environmental stress such as extreme temperatures and pH or oxidative stress can lead to protein aggregation [2]. In addition, defective cellular protein quality-control system that refolds or degrades misfolded proteins is another important factor in protein aggregation [2].

α-Syn aggregates are key molecules of synucleinopathies including PD, dementia with Lewy bodies, and multiple system atrophy [4]. Multiplications of the gene encoding α-syn (SNCA) [5] and various point mutations in this gene (A30P, E46K, and A53T) [6–8] contribute to dominant familial parkinsonism by enhancing the propensity to aggregation [9]. Environmental factors (*i*.*e*. oxidative stress), post-translational modifications, and proteolysis are shown to induce aggregation of α-syn [10, 11]. α-Syn, as a neuronal presynaptic protein, modulates synaptic transmission by playing different roles according to its form [12]. As a monomer, α-syn has a physiological role in the regulation of neurotransmitter release, synaptic function, and plasticity [12]. Also, α-syn involves the trafficking of synaptic vesicles and in the regulation of vesicle exocytosis, and thus controls vesicle homeostasis [13–15]. However, α-syn aggregates, as a main pathogenic factor, cause cytotoxicity by affecting many cellular mechanisms such as damaging mitochondria and proteasome, blocking endoplasmic reticulum-Golgi trafficking, triggering lysosomal leakage or disrupting microtubules [16–19]. Moreover, α-syn aggregates mediate synaptic dysfunction by interfering with the axonal transport of synaptic proteins such as Synapsin 1 or inhibiting synaptic vesicle reclustering and thus reduce neurotransmitter release [20, 21]. Abnormal accumulation of α-syn aggregates are initially found in dopaminergic neurons of substantia nigra but gradually propagate to neocortex by cell-to-cell transmission via the mechanisms of endocytosis, direct penetration, trans-synaptic transmission or membrane receptors [22–27].

*In vitro* studies have shown that diverse and heterogeneous α-syn oligomeric species are generated under different conditions and implicated in the α-syn toxicity occurring through different mechanisms [28, 29]. Different morphological species of α-syn including spherical, chain-like, rod-like and annular forms have been exhibited and the multifunctional properties of α-syn aggregates are based on its conformational flexibility [30, 31]. Spherical oligomers have been reported to induce abnormal calcium currents by affecting membrane permeability in cultured primary cortical neurons and finally result in neuronal degeneration [32]. Short rod-shaped oligomers inhibit SNARE-mediated vesicle docking accompanied by reduced exocytosis [33]. These oligomers are formed transiently in the process of aggregation and possess different physical properties leading to cell damage directly or indirectly [34–36]. One of the main characteristics of toxic oligomers is perturbation of the biological membrane and consequent disruption of cellular function [32].

In this study, we show that α-syn aggregates sequester functional synaptic protein such as VAMP2 in the diverse neuronal systems. The sequestration of cellular proteins by α-syn aggregates led to neurotoxicity and subsequently cell death. Moreover, we identified a VAMP2-derived specific peptide that inhibited VAMP2 sequestration by α-syn aggregates, thus contributing to a prevention of α-syn aggregates-mediated cellular toxicity. Therefore, our findings provide a mechanism by which α-syn aggregates impact neuronal toxicity and a potential therapeutic drug development in PD.

## Materials and methods

### Preparation of recombinant proteins

Recombinant human VAMP2 was expressed in *Escherichia coli* Rogetta^TM^(DE3)pLysS (Novagen, USA) cell strain transformed with a pET28a plasmid containing the soluble VAMP2 sequence. The cells were grown at 37°C in LB medium with 50 µg/ml kanamycin until the absorbance at 600 nm reached 0.6–0.8. Isopropyl β-D-1-thiogalactopyranoside (0.5 mM final concentration) was added and the cells were incubated for overnight at 16°C. The cells were sonicated in lysis buffer (25 mM HEPES, pH 7, 300 mM KCl, 20 mM imidazole, and 1X protease inhibitor cocktail) and centrifuged at 15,000 g for 30 min at 4°C. The cell lysate was mixed with Ni-NTA agarose (Qiagen, Germany) for 1 h at 4°C. After binding, the beads were washed extensively with wash buffer (25 mM HEPES, pH 7, 300 mM KCl, and 20 mM imidazole). The proteins were eluted in elution buffer (25 mM HEPES, pH 7, 300 mM KCl, and 400 mM imidazole) and subjected to dialysis. Recombinant human Syntaxin1A (1–226) and SNAP25 were purchased from ProSpec Biotechnology. Recombinant human α-syn was purified as previously described [37] at Daegu-Gyeongbuk Medical Innovation Foundation (Daegu, Korea). The purity of all recombinant proteins was confirmed by SDS-PAGE (S1 Fig).

### Preparation of protein aggregates

α-Syn aggregates showing spherical shape were generated as previously described [33]. Purified 10 µM α-syn was incubated with 200 µM dopamine in 20 mM sodium phosphate buffer (pH 7) for 3 days at 37°C with constant agitation. The sample was centrifuged at 14,000 g for 10 min at 4°C to remove insoluble aggregates. The supernatant was concentrated and loaded on a Superdex 200 10/30 GL column (GE Healthcare, UK) to separate aggregates from monomer using PBS in NGC^TM^ Chromatography Systems (Bio-Rad, USA). Fractions containing aggregates were concentrated again and stored at 4°C until use. Another spherical form of aggregates was prepared as described previously [38]. Briefly, α-syn (12 mg/ml) was incubated in PBS at 37°C for 24 h without agitation. The excess of monomeric protein and the low levels of oligomer were separated by size-exclusion chromatography. α-Syn preformed fibrils (PFF) were prepared by agitating α-syn (5 mg/ml) in PBS at 37°C. After 7 days of incubation, α-syn aggregates were sonicated for 30 s and the monomer and PFF were separated by size-exclusion chromatography. The fractions containing monomer and PFF were kept at -80°C. To generate Aβ aggregates, Aβ_1–42_ peptide (GenScript, USA) was dissolved in HPLC grade water at 1 mg/mL and 10 µM peptide was incubated in PBS at 37°C for 5 days.

### Transmission electron microscopy analysis

Samples were loaded on glow discharged 200-mesh copper grids (Electron Microscopy Sciences, USA) and negatively stained with 2% (w/v) uranyl acetate. The images were obtained with Tecnai G2 transmission electron microscope (FEI, USA).

### Cell-free protein binding assay

1 µM recombinant proteins were incubated with 100 nM α-syn aggregates for 2 h at 37°C in a total volume of 10 µl. Reactions were stopped by adding 5X protein sample buffer and then subjected to SDS-PAGE without boiling to maintain the integrity of aggregates. Protein levels were detected by western blot analysis or Sypro^®^ Orange (Molecular Probes, USA) protein gel stains. For quantification of protein band intensity, gel analysis software Bio-1D (Vilber Lourmat, Germany) was used.

### Western blot analysis

Cells were extracted with ice-cold Nonidet P-40 lysis buffer (50 mM Tris, pH 7.5, 150 mM NaCl, 30 mM MgCl_2_, 1% Nonidet P-40, 1 mM DTT) and a mixture of protease and phosphatase inhibitors. Lysates were then centrifuged for 10 min at 14,000 g at 4°C. 30 µg of protein from each supernatant was subjected to 12% SDS-PAGE and transferred to a nitrocellulose membrane which was blocked with 3% BSA for 1 h at room temperature. Immunoblotting was performed with primary specific antibodies listed in S1 Table. The peroxidase-conjugated secondary antibody was incubated for 1 h at room temperature and developed with enhanced chemiluminescence using Fusion FX7 (Vilber, Germany).

### Primary neuronal culture

Primary cortical neurons were cultured from embryonic day 18 rat brains and incubated in neurobasal media supplemented with B-27, 0.5 mM L-glutamine, 12.5 µM glutamate, 100 units/mL penicillin and streptomycin (all from Invitrogen, USA) on tissue culture plates coated with poly-D-lysine (Sigma, USA) [39]. The neurons were maintained by changing medium (without glutamate) every 3–4 days. All experiments were performed at 7–14 DIV. 5 µg/mL of α-syn aggregates were added to a 12-well plate and 1 µg/mL to a 96-well plate. For peptide experiments, 1 mM peptide was pre-incubated with α-syn aggregates for 1 h at room temperature and treated to the cells. For transfection experiment, Pro-Ject^TM^ Protein Transfection Reagent (Pierce, USA) was used according to the manufacturer’s instructions.

### Cell culture

Human neuroblastoma SH-SY5Y (ATCC^®^ CRL-2266^™^) cells were cultured at 37°C in humidified air with 5% CO_2_ in Dulbecco’s modified Eagle’s medium containing 10% fetal bovine serum, 100 units/ml penicillin and streptomycin. SH-SY5Y cells were differentiated by treatment of the cells with 15 µM retinoic acid (Sigma, USA) every 2 days for 1 week.

### Animals

Sprague Dawley rats were purchased from OrientBio (Korea) and used for the primary neuronal culture as described above after euthanasia by CO_2_ inhalation. Prnp-SNCA*A53T transgenic mice [40] were purchased from The Jackson Laboratory (Stock No. 004479) and maintained on C57BL/6 and C3H mixed background. A maximum 5 adult mice in one cage were maintained on a 12 light/12 dark cycle in the specific pathogen free facility. This study was carried out in strict accordance with the recommendations in the Guide for the Care and Use of Laboratory Animals of the National Institutes of Health. The protocol was approved by the Institutional Animal Care and Use Committee of Korea Brain Research Institute (IACUC-17-00002, IACUC-17-00012). All animals were immediately sacrificed by cervical dislocation or CO_2_ inhalation to minimize suffering.

### Immunofluorescence staining

For immunocytochemistry, neurons were fixed with 4% paraformaldehyde in PBS followed by permeabilization with 0.1% Triton X-100 in PBS. Neurons were incubated with primary specific antibodies (detailed in S1 Table) followed by fluorescent secondary antibodies conjugated to Alexa-fluor 488 or 568 (Invitrogen, 1:1000). For neuronal images, images were taken at 20X with 4X software magnification. For immunohistochemistry, brains postfixed in 4% paraformaldehyde followed by a sucrose cryoprotection were sliced at 60 µm on a freezing microtome. Brains were incubated with primary antibodies (detailed in S1 Table) in 5% goat serum PBST (0.3% Triton X-100) overnight at 4°C, followed by incubation with fluorescent-conjugated secondary antibodies for 2 h at room temperature. All sections were mounted onto slides and coverslipped using ProLong^TM^ Gold mounting solution (Invitrogen, USA). Confocal microscopy data were acquired with confocal laser scanning microscopy TCS SP8 (Leica, Germany) at the Advanced Neural Imaging Center in Korea Brain Research Institute.

### *In vivo* pull-down assay

Young (3 months) and old (22 months) heterozygous SNCA mice were sacrificed by the cervical dislocation and brain tissues were homogenized in ice-cold lysis buffer (T-PER^®^ tissue protein extraction reagent, ThermoFisher Scientific, USA) containing 1X proteinase inhibitor cocktail. Homogenized tissues were incubated in ice for 10 min and centrifuged at 10,000 g for 5 min at 4°C. The protein concentration of brain lysate was determined by Bicinchoninic acid (BCA) protein assay (ThermoFisher Scientific, USA). 3 mg of brain lysates were immunoprecipitated with Sepharose beads 4B (GE Healthcare, UK) pre-incubated with anti-VAMP2 antibody (Abcam, USA) at 4°C overnight, then immunoblotted using anti-α-syn antibody (BioLegend, 1:1000).

### Peptide synthesis

VAMP2 peptides were synthesized by PEPTRON (Daejeon, Korea) with purity greater than 95%. The peptides were dissolved in water at the final concentration of 10 mg/ml and stored in appropriate aliquots at -20°C until analysis.

### Glutamate release assay

Released glutamate levels were measured using glutamate assay kit (Cell Biolabs, USA). Briefly, insoluble particles were removed by centrifugation at 10,000 rpm for 5 min and the supernatant was assayed directly in FlexStation 3 (Molecular Devices, USA). Glutamate levels were quantified based on the Glutamate standard curve. All other procedures were followed by manufacturer’s instruction.

### Cell viability assay

Neurons were plated in 96-well plates. Post α-syn aggregates (1 µg/mL) treatment 14 days, cell viability was measured using CellVia cell viability assay kit (AbFrontier, Korea). CellVia solution was added (10 µl/well) to the wells and incubated for 4 h at 37°C. The absorbance was measured on VersaMax microplate reader (Molecular Devices, USA) at 450 nm.

### FM 1–43 imaging

Vesicular recycling in SH-SY5Y cells was visualized with an FM1-43 FX dye (Invitrogen, USA). 5 µg/ml FM1-43 FX dye solution was prepared in HEPES-buffered saline solution (HBSS) containing 118 mM NaCl, 5.4 mM KCl, 10 mM glucose, 10 mM HEPES (pH 7.4), 1.2 mM MgSO_4_ and 2 mM CaCl_2_. The coverslips were removed from the culture medium and rinsed with HBSS. Then, coverslips were immersed in FM1-43 FX dye solution for 90 s at room temperature. After rinsing three times with HBSS on the ice, the stain was fixed with ice-cold 4% paraformaldehyde for 20 min. The coverslips were rinsed three times and then mounted with ProLong^TM^ Gold antifade reagent (ThermoFisher Scientific, USA).

### ROS measurement

Intracellular ROS levels were determined using 2′,7′-dichlorofluorescin diacetate (DCFDA) (Sigma, USA). Subconfluent differentiated SH-SY5Y cells cultured on 96-well plate were incubated with α-syn aggregates pre-incubated with or without P5. After 5 h treatment, the cells were loaded with 5 µM DCFDA for 30 min at 37°C, washed with HBSS and measured at 488 nm in FlexStation 3 (Molecular Devices, USA).

### LDH cytotoxicity assay

LDH release was measured using Pierce LDH Cytotoxicity Assay Kit (ThermoFisher Scientific, USA) in VersaMax microplate reader (Molecular Devices, USA). All procedure was followed by manufacturer’s instruction.

### Calcium assay

Intracellular calcium levels were measured with the fluorescent calcium indicator, Fluo-4 acetoxymethyl (AM) ester (ThermoFisher Scientific, USA). Fluo-4 AM diluted in neuronal media was added to the neuronal cells and incubated for 30 min at 37°C in a humidified atmosphere (5% CO_2_, 95% air). After 30 min incubation, dye solution was removed and cells were washed with PBS. Fluorescence was measured at 488 nm in FlexStation 3 (Molecular Devices, USA).

### Statistical analyses

Graphs were obtained with GraphPad Prism and statistical analyses were performed with Student’s *t*-test for comparison of two different groups and repeated-measures analysis of variance or one-way analysis of variance (ANOVA) for more than two different groups. The error bars represent the SEM.

## Results

### α-Syn aggregates sequester specific synaptic proteins

**Fig 1:**
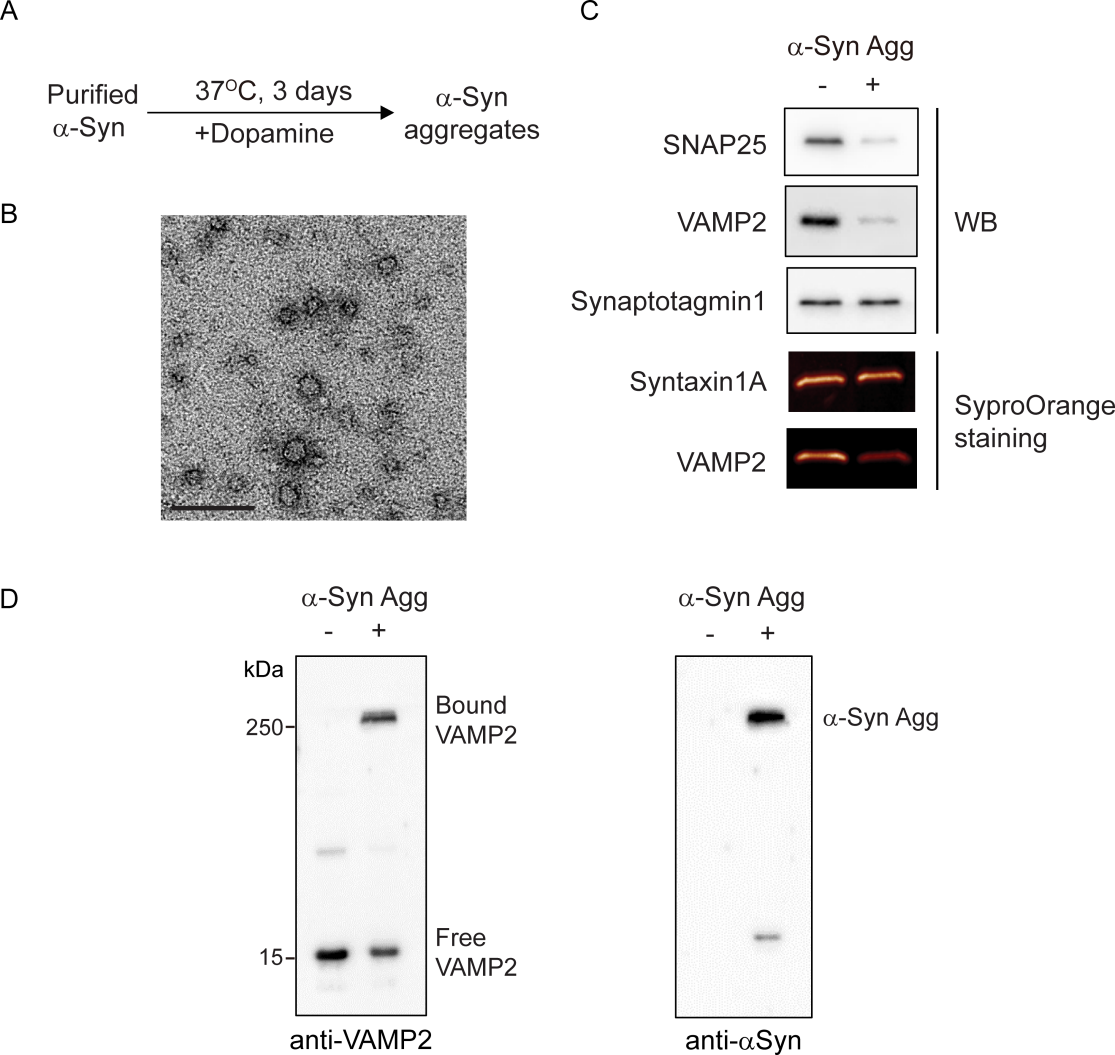
α-Syn aggregates reduce selectively synaptic protein levels in cell-free systems. (A) α-Syn aggregates were generated by incubation with dopamine at 37°C for 3 days followed by size-exclusion chromatography. (B) Transmission electron micrograph of α-syn aggregates showed the spherical shape and 10–20 nm in diameter. Scale bar, 100 nm. (C) Recombinant synaptic proteins (1 µM) were incubated with α-syn aggregates (100 nM) at 37°C for 2 h and subjected to western blot analysis or SyproOrange staining to detect protein levels. (D) Recombinant VAMP2 was incubated with α-syn aggregates at 37°C for 2 h. Western blot analysis using anti-VAMP2 (left) or anti-α-syn antibody (right), respectively showed a strong binding of VAMP2 to α-syn aggregates.

To investigate biochemical mechanism of synaptic dysfunction mediated by oligomeric α-syn aggregates [20, 22, 33], we first generated α-syn aggregates by the dopamine-induction method, which is known to accelerate and stabilize oligomeric form of α-syn, but inhibits the process of fibril formation [33, 41–43] (Fig 1A). Transmission electron microscopy (TEM) analysis revealed that the purified α-syn aggregates according to this procedure were a spherical shape with a size of 10–20 nm in diameter (Fig 1B). In aggrement with the previous study [42], dopamine-mediated α-syn aggregates were thioflavin T-negative (data not shown) and non-fibrillar species. Then, purified α-syn aggregates were incubated with some recombinant synaptic proteins to test their direct interaction. Interestingly, specific SNARE components such as SNAP25 and VAMP2 were greatly reduced in the presence of α-syn aggregates, whereas Syntaxin1A and Synaptotagmin1 did not change (Fig 1C). This result indicated that the oligomeric α-syn aggregates are able to preferentially interact with specific SNARE components. In particular, VAMP2 exhibited strong SDS-resistant binding to α-syn aggregates on western blot analysis (Fig 1D) that was confirmed by superimposed image (S2 Fig). We also observed that SNAP 25 bound to α-syn aggregates directly (S2 Fig).

**Fig 2:**
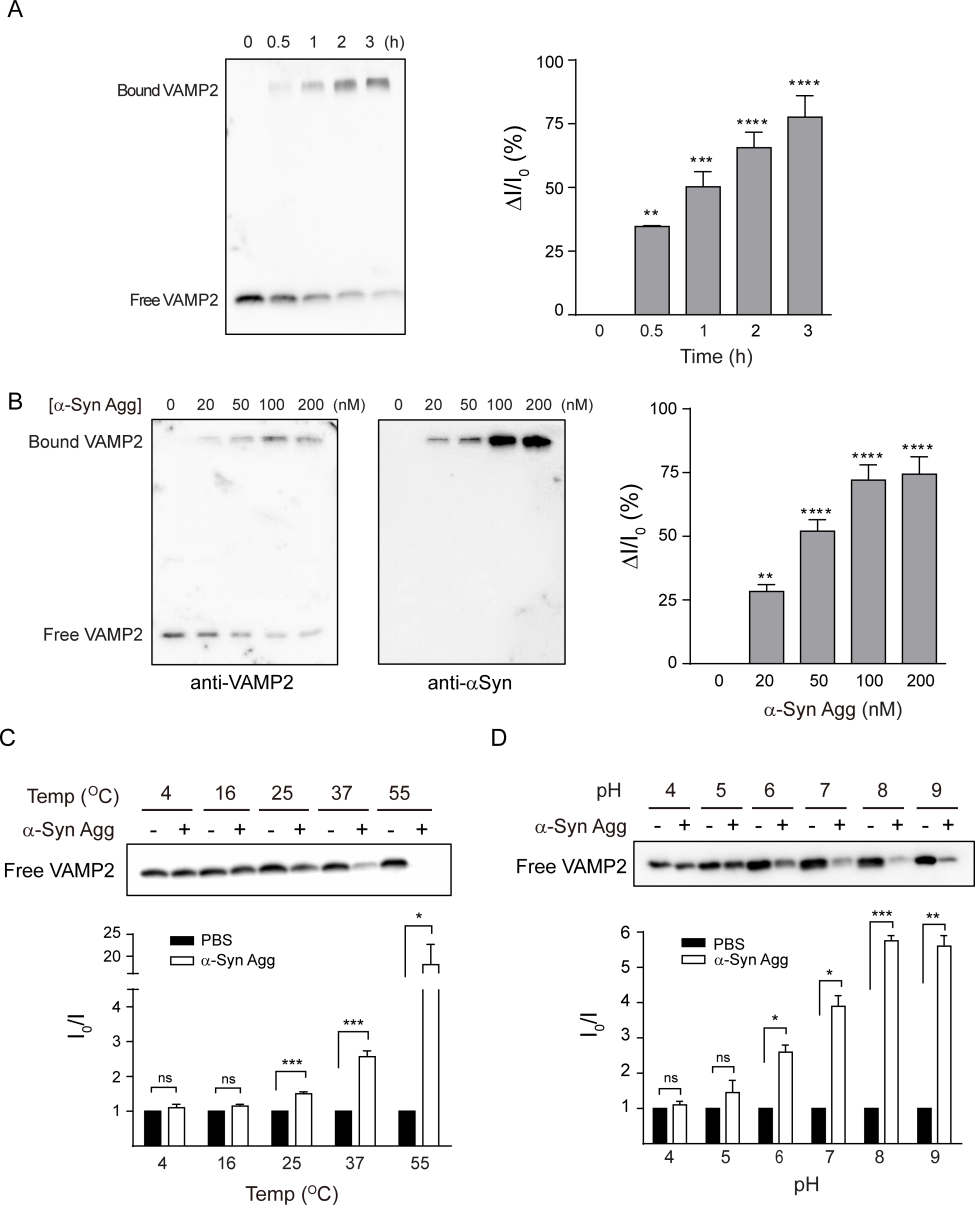
α-Syn aggregates sequester VAMP2 through direct binding *in vitro*. (A) VAMP2 was incubated with α-syn aggregates (100 nM) at 37°C for the indicated time points. ΔI/I_0_ values indicate the relatively bound VAMP2 protein levels, where I_0_ is the intensity of control free VAMP2 at time 0 and I is the intensity of each free VAMP2 at the different time point (ΔI = I_0_-I). (B) VAMP2 was incubated with different concentration of α-syn aggregates at 37°C for 2 h. I_0_ is the intensity of control free VAMP2 and I is the intensity of each free VAMP2 at different concentration of α-syn aggregates. (C-D) The binding assay was performed at different temperature and pH. I_0_ and I are the intensity of free VAMP2 in the absence and presence of α-syn aggregates, respectively, at the indicated temperature and pH. Each blot is a representative immunoblot from two or three independent experiments. *, p < 0.05, **, p < 0.01, ***, p < 0.001, by two-tailed, unpaired *t*-test. Values indicate mean ± SEM.

For a further mechanistic study of the specific target selection of α-syn aggregates, we focused on VAMP2 since this protein is known to bind with α-syn among the SNARE complex components [33, 44, 45]. Furthermore, it is previously reported that VAMP2 is more susceptible to neurotoxins than the other binding target, SNAP25 [46, 47], suggesting that VAMP2 could be easily impaired by α-syn aggregates. To further characterize the biochemical properties of VAMP2 binding to α-syn aggregates, we performed a set of the binding assay in different cell-free conditions. As the incubation time increased, the protein levels of free VAMP2 decreased and those of bound VAMP2 clearly increased (Fig 2A). The binding of VAMP2 to α-syn aggregates was also dependent on the concentration of α-syn aggregates (Fig 2B). In addition, the binding capacity was enhanced in a broad range of temperature and pH that covers physiological condition (Fig 2C and 2D).

**Fig 3:**
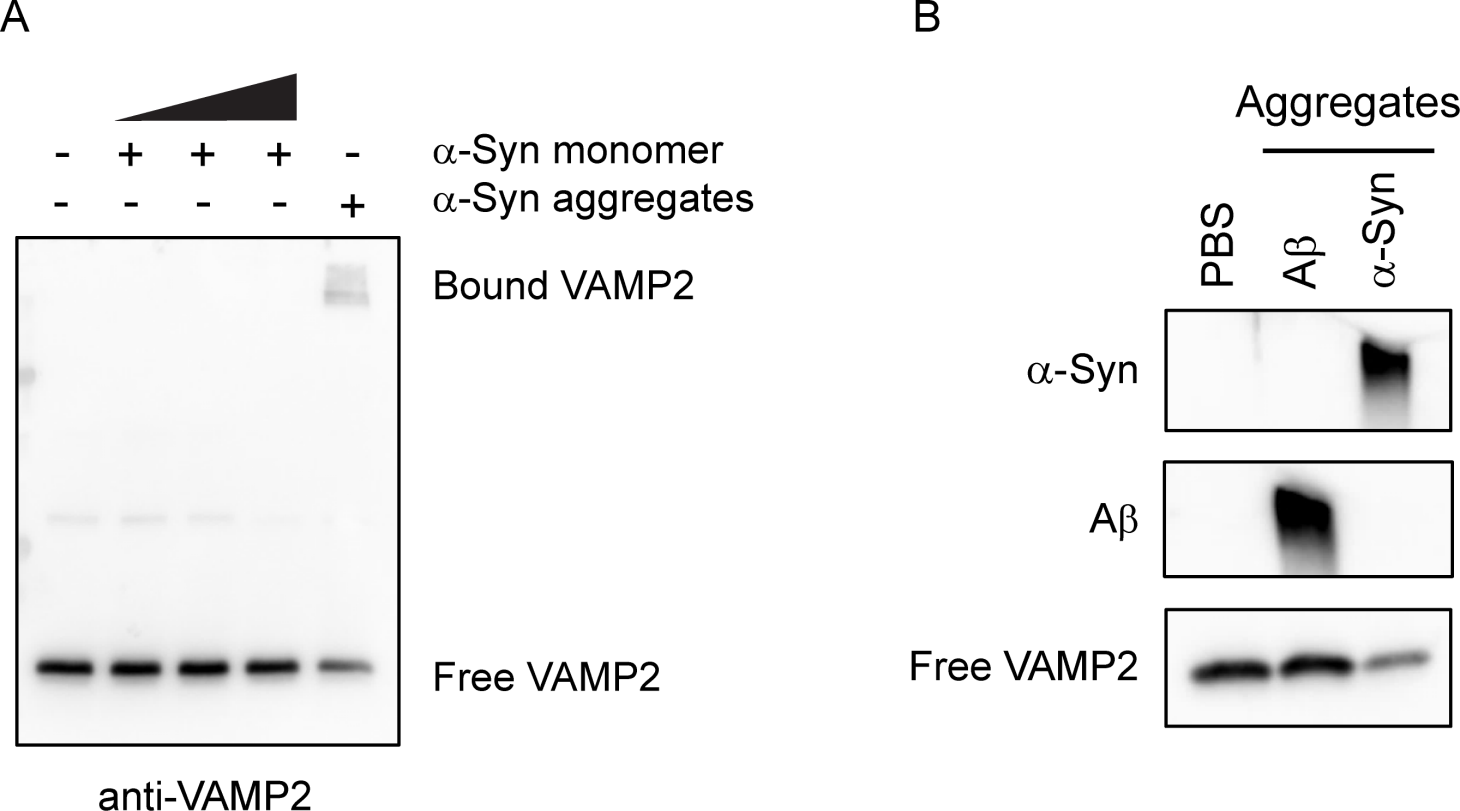
VAMP2 does not bind to α-syn monomer and Aβ aggregates. Recombinant VAMP2 was incubated with the increasing concentration (0.1, 1, 10 µM) of α-syn monomer (A) or 100 nM Aβ aggregates (B) at 37°C for 2 h. Western blot analysis shows no binding of VAMP2 to α-syn monomer or Aβ aggregates. The weak band intensity of bound VAMP2 to α-syn aggregates was due to the saturated signals from free VAMP2.

To investigate whether the specific binding occurs solely to α-syn aggregates, monomeric form of α-syn was incubated with VAMP2 (Fig 3A). However, VAMP2 did not bind to α-syn monomer even in much higher concentration. Likewise, to rule out the possibility that VAMP2 protein *per se* tends to bind to any pathological protein aggregates, amyloid β (Aβ) aggregates were tested in the same binding condition (Fig 3B). We could not observe VAMP2 binding to Aβ aggregates either, suggesting that the specific binding to VAMP2 is a unique characteristic of α-syn aggregates. Taken together, our results accompanying the previous findings [45] clearly demonstrate that α-syn aggregates sequester synaptic proteins such as VAMP2 through direct and specific interaction mechanism.

Even though dopamine-induced α-syn aggregates is relatively stable and highly homogenous (S3 Fig), it is possible that dopamine oxidation may cause the specific VAMP2 binding. To rule out this possibility, we generated α-syn aggregates in the absence of dopamine and compared the relative effectiveness on the VAMP2 binding. First, the spherical form of aggregates was generated without dopamine as described previously [38], however, the efficiency of aggregation was very low (S4 Fig). Despite such a low aggregation, we could observe the low level of VAMP2 binding on aggregates (S4 Fig). Next, since synthetic α-syn preformed fibrils (PFF) have been widely reported as a pathological form of α-syn aggregates [22, 37, 48, 49], the pre-fibrillar form of α-syn was generated as reported previously with modification [37] and its shape was confirmed using TEM analysis (S5 Fig). Interestingly, VAMP2 was also bound to PFF like spherical aggregates, suggesting that both types of aggregates were able to sequester VAMP2 (S5 Fig). These results indicated that dopamine was not involved in the interaction. Then, to compare the relative cellular toxicity induced by these different forms of aggregates, intracellular calcium levels were measured in primary cultured neuronal cells since calcium influx is increased in response to α-syn PFF [49]. Treatment of both α-syn spherical aggregates and PFF led to increased intracellular calcium levels similarly (S5 Fig). α-Syn aggregates, hereinafter, referred to the dopamine-induced spherical shape of aggregates.

### α-Syn aggregates sequester VAMP2 protein in the cultured neurons as well as PD mouse model

**Fig 4:**
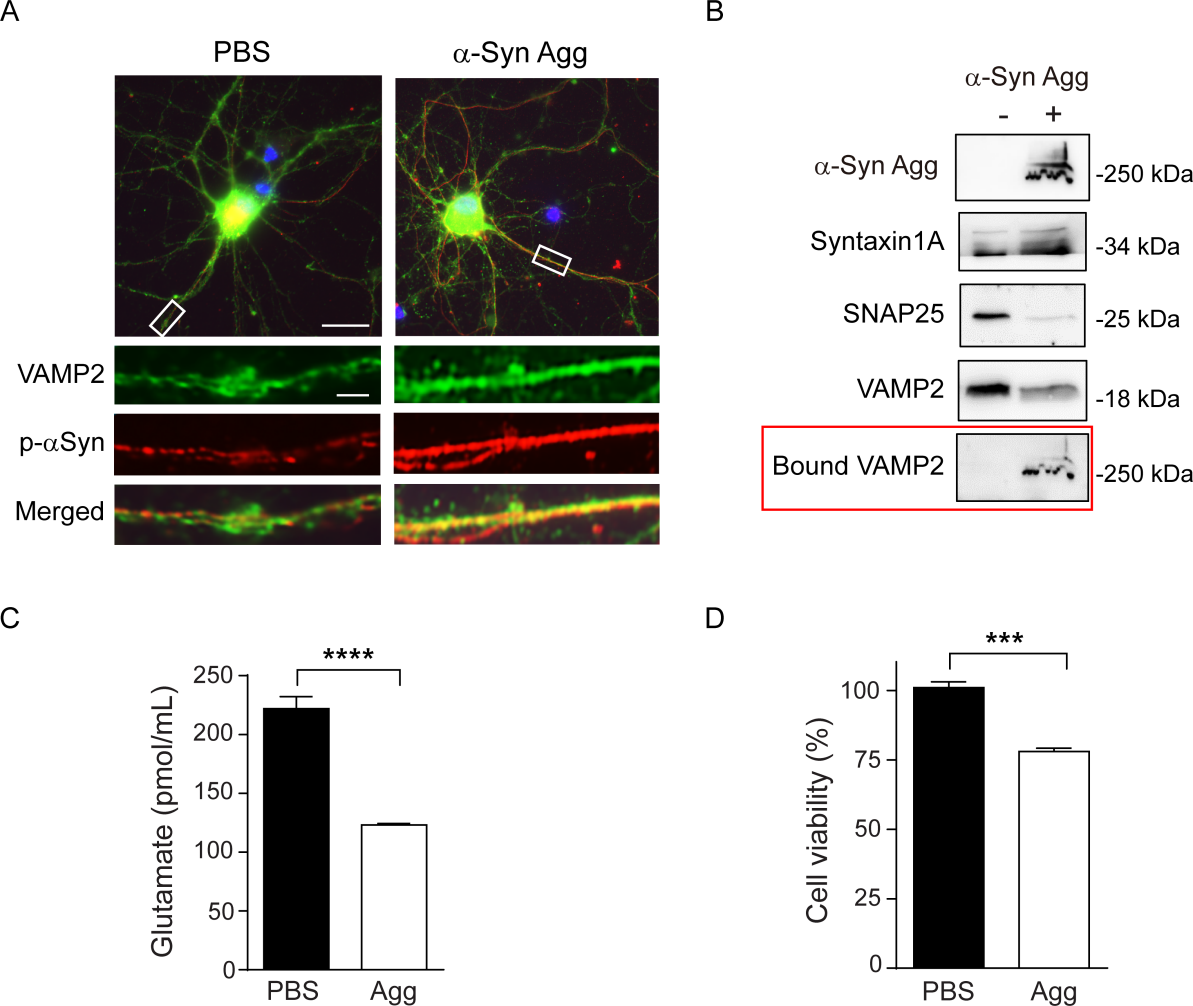
VAMP2 sequestration by α-syn aggregates in cultured primary neurons results in synaptic dysfunction as well as cytotoxicity. (A) Immunocytochemistry in rat cortical primary neurons shows a direct binding of VAMP2 to α-syn aggregates. (B) Western blot analysis of cultured neurons shows selectively reduced VAMP2 and SNAP25 levels in α-syn aggregates-treated cells for 3 days consecutively. To analyze bound VAMP2, the blot used for α-syn aggregates detection was re-probed, marked in the red box. (C) Cortical neuronal cells were treated with α-syn aggregates for 3 days consecutively and glutamate release was determined. ****, p < 0.0001, by two-tailed, unpaired *t*-test (n = 10). (D) Cell viability was determined 14 days after addition of α-syn aggregates in cortical neurons. ***, p < 0.001, by two-tailed, unpaired *t*-test (n = 3). Values indicate mean ± SEM.

Next, we examined the effect of α-syn aggregates on VAMP2 in primary cultured neuronal cells. After incubation of α-syn aggregates in the cultured rat cortical neurons for 7 days, direct binding between VAMP2 and α-syn aggregates was clearly observed (Fig 4A). To examine the effect of α-syn aggregates on cellular synaptic protein levels, α-syn aggregates were treated to neuronal cells and western blot analysis was performed. As shown in Fig 4B, VAMP2 and SNAP25 protein levels were significantly reduced in the α-syn aggregates-treated cells compared to non-treated cells, while Syntaxin1A did not change in protein level. In addition, we also observed bound VAMP2 to α-syn aggregates (Fig 4B, red box) in the same western blot. These results confirmed the sequestration effect of α-syn aggregates to the preferential target proteins in neuronal cells and strongly supported the aforementioned our findings obtained from the cell-free system.

To assess the sequestration effect by α-syn aggregates on synaptic functions, neurotransmitter release was determined by measuring released glutamate levels in neuronal cells after α-syn aggregates treatment for 3 days. Glutamate release was remarkably decreased by ~45% in the α-syn aggregates-treated cells (Fig 4C). Also, the neurons exposed to α-syn aggregates for 14 days showed reduced cell viability, suggesting that neuronal dysfunction through the direct sequestration in cellular levels possibly led to serious impairment of cell survival (Fig 4D).

**Fig 5:**
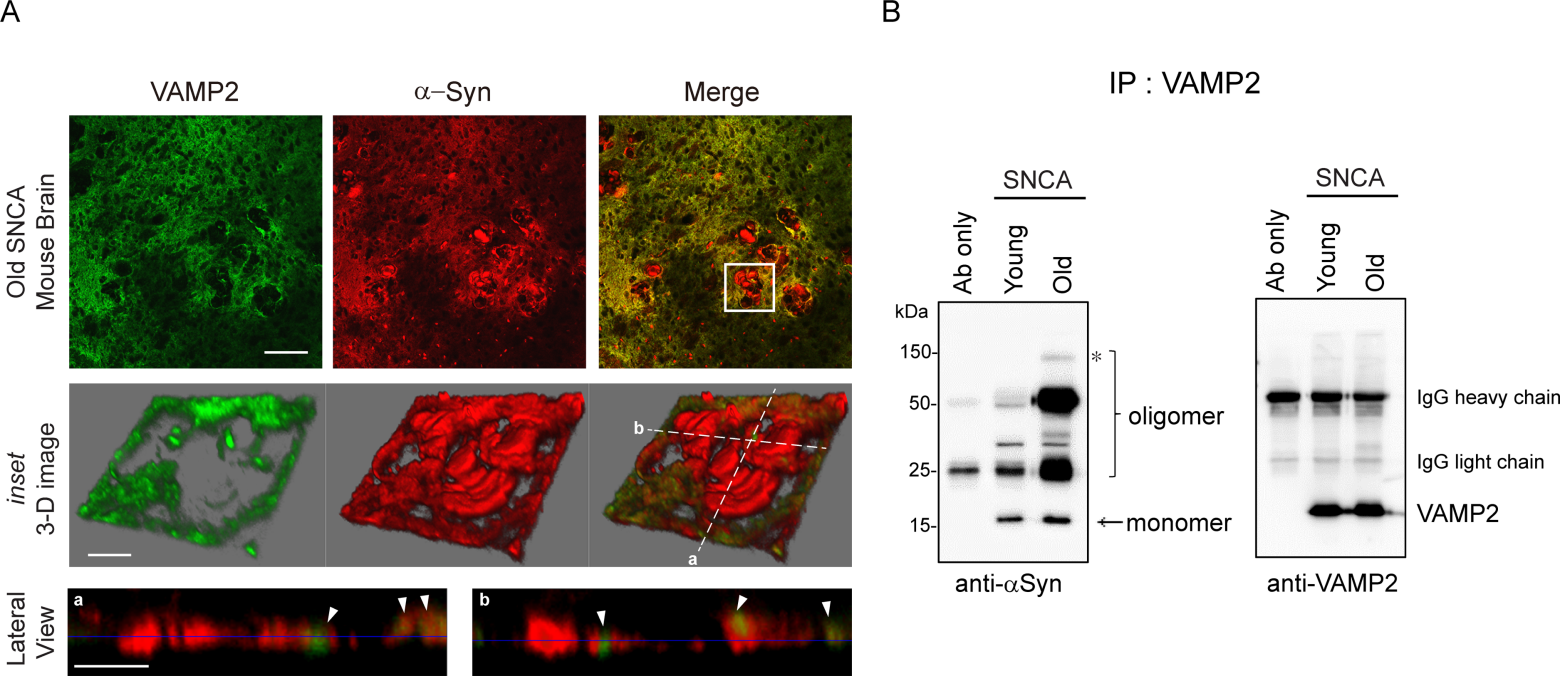
α-Syn aggregates from PD mouse model directly bind to VAMP2 *in vivo*. (A) Immunohistochemistry of basal ganglia from 22 months old SNCA mouse brain. Multiple α-syn aggregates (distinct red signals in the upper right panel) are shown in the old SNCA mouse brain and one of the aggregates (white boxed inset) is shown at a high magnification 3-dimensional image in the middle panels. Two lateral section images crossed in white dotted lines (a and b from the middle right panel) are displayed in the bottom panels. The arrowheads in white indicate the clustered VAMP2 in the α-syn aggregates. Scale bars (upper panels; 100 µm, middle and lower panels; 20 µm) (B) Young (3 months) and old (22 months) SNCA mice brain tissue lysates were subjected to immunoprecipitation using VAMP2 antibody followed by immunoblot using α-syn antibody. Oligomer species were increased in old SNCA mice brain (marked in bracket) and high molecular weight oligomer (marked an asterisk) was detected only in the old mice. Immunoblot of VAMP2 was used as loading control. Immunoblot is a representative blot from two independent experiments.

To further verify the sequestration of VAMP2 by α-syn aggregates *in vivo* system, we used young (3 months) and old (22 months) heterozygous A53T α-syn transgenic mice (hereinafter referred as SNCA), which carries human α-syn with the A53T mutation under the mouse prion protein promoter [40]. As expected, old SNCA mice showed obvious α-syn pathologies (Fig 5A, inset) such as apparent α-syn aggregation and VAMP2 clustering adjacent to the α-syn aggregates (Fig 5A, lateral view images). Besides, an excessive amount of α-syn oligomeric forms was co-precipitated with VAMP2 in the old SNCA mice compared to the young mice (Fig 5B). In particular, we also noticed that high molecular weight oligomeric form of α-syn was detected only in the old mice (Fig 5B, asterisk). All these results strongly supported that age-dependent increase in α-syn oligomers and their interaction with VAMP2 may cause synaptic failure and ultimately develop PD symptom [29].

### Identification of small peptide to interfere VAMP2-α-syn aggregates interaction

**Fig 6:**
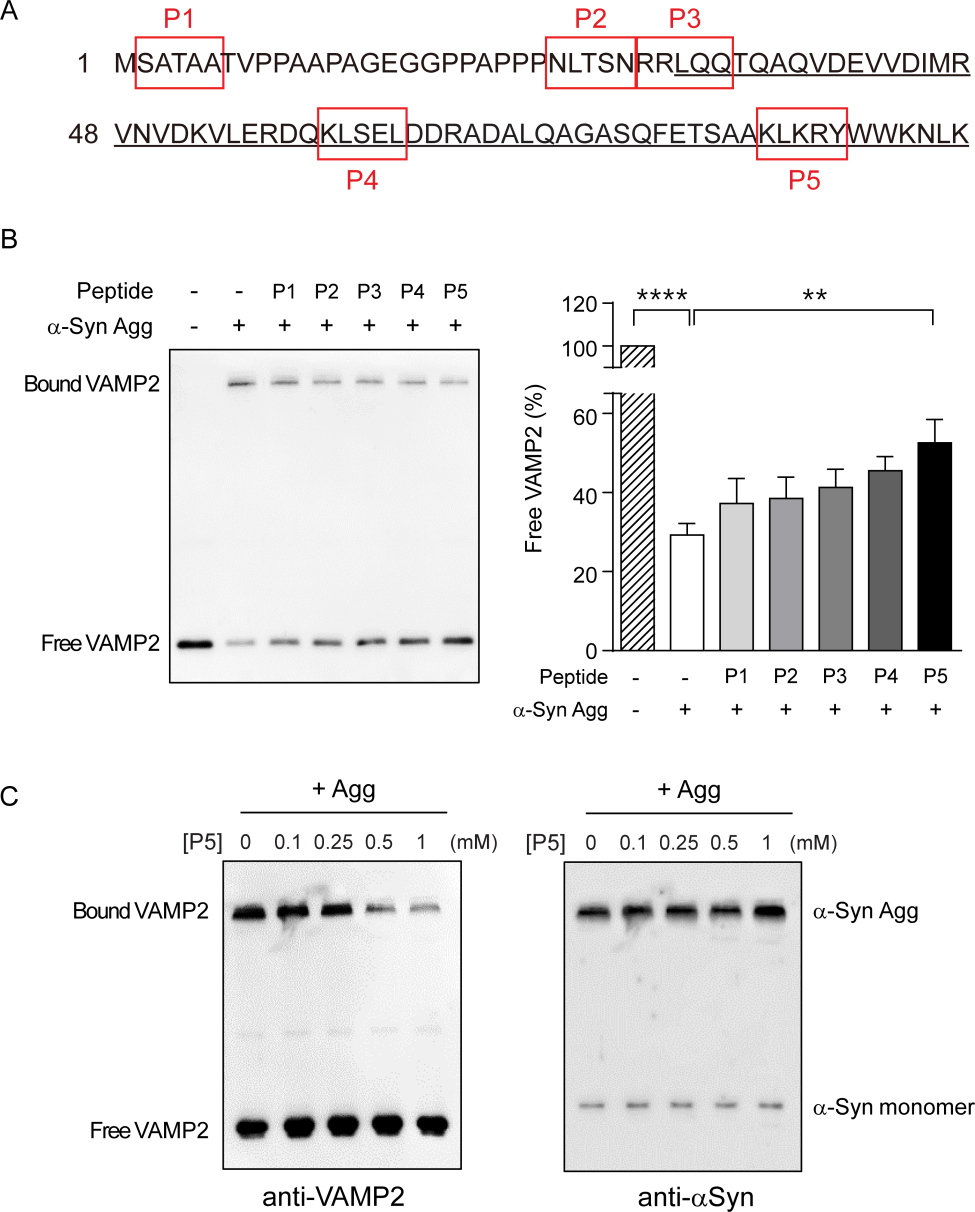
VAMP2 peptide partially abolishes VAMP2 sequestration by α-syn aggregates in the cell-free conditions. (A) Five different VAMP2 peptides designated P1 to P5 in red boxes were designed based on the sequence of a soluble part in VAMP2. SNARE motif was underlined. (B) α-Syn aggregates and the indicated peptides (1mM) were pre-incubated for 1 h at room temperature and then VAMP2 binding assay was performed. The bar graph shows the free VAMP2 levels in the presence of α-syn aggregates and each peptide. Error bars were obtained from four independent experiments. ****, p < 0.0001, **, p < 0.01 by ANOVA with Dunnett’s multiple comparisons test. Values indicate mean ± SEM. (C) The VAMP2 binding assay was performed in the presence of α-syn aggregates pre-incubated with the indicated concentrations of peptide P5. VAMP2 sequestration by α-syn aggregates was inhibited by peptide P5 in a dose-dependent manner (left). Free VAMP2 levels seemed to reach the saturation with a minor increase. Immunoblot is a representative blot from three independent experiments.

The specific binding between α-syn aggregates and VAMP2 reflects the presence of interacting domain in the VAMP2 protein. Therefore, we sought to identify small peptide-inhibitors which interfere with the VAMP2-α-syn aggregates interaction. Previously, a small peptide containing only 4 residues has been shown to play a role as a substrate inhibitor in the binding of SNAP25 to Botulinum neurotoxin [47]. Thus, we designed several synthetic small peptides based on the sequence of VAMP2 to block the binding of VAMP2 to α-syn aggregates (Fig 6A, red box). Since the N-terminal region of VAMP2 was known to interact with the C-terminus of α-syn monomer [44], we predicted that the N-terminal peptides (*i*.*e*. P1 or P2) might be able to block the binding. Unexpectedly, however, peptide P5 (^83^KLKRY^87^) derived from the C-terminal region of SNARE motif in VAMP2 inhibited the binding the most efficiently among the tested peptides by showing the 2-fold increase in free VAMP2 level (Fig 6B). In the same cell-free condition, peptide P5 inhibited VAMP2 binding in a dose-dependent manner in the presence of α-syn aggregates (Fig 6C, left). Additionally, we excluded the possibility that α-syn aggregates were disaggregated by peptide P5 since α-syn aggregates levels were maintained in spite of increasing peptide P5 concentration (Fig 6C, right). We also investigated if peptide P5 can block SNAP25 binding to aggregates and observed that peptide P5 efficiently prevented the sequestration of SNAP25 in the cell-free system (S6 Fig).

### VAMP2 small peptide protects cells from α-syn aggregates-mediated toxicity

Next, we assessed whether peptide P5 could rescue α-syn aggregates-mediated cytotoxicity in various aspects. First, we examined the effect of peptide P5 on glutamate release. However, we were not able to observe the statistically significant preventive role of peptide P5 (S7 Fig). This result suggested that peptide P5 may not fully protect all functional synaptic components damaged by α-syn aggregates. Nonetheless, peptide P5 itself did not affect glutamate release (S7 Fig) indicating that peptide P5 itself may not influence SNARE function despite its origin from VAMP2. Although we did not observe a preventive role of peptide P5 in synaptic function as described above, we noticed that the neurons co-treated with peptide P5 showed less cytotoxicity in response to α-syn aggregates (S7 Fig). These observations led us to consider the possibility that peptide P5 may positively affect general cellular functions. Therefore, we next examined whether peptide P5 was effective in reducing cellular toxicity caused by α-syn aggregates through multiple analyses using human neuroblastoma SH-SY5Y cells and rat primary cortical neurons. Considering short half-life of the peptide in solution, we examined the functional effect of peptide P5 in cells within a short period.

**Fig 7:**
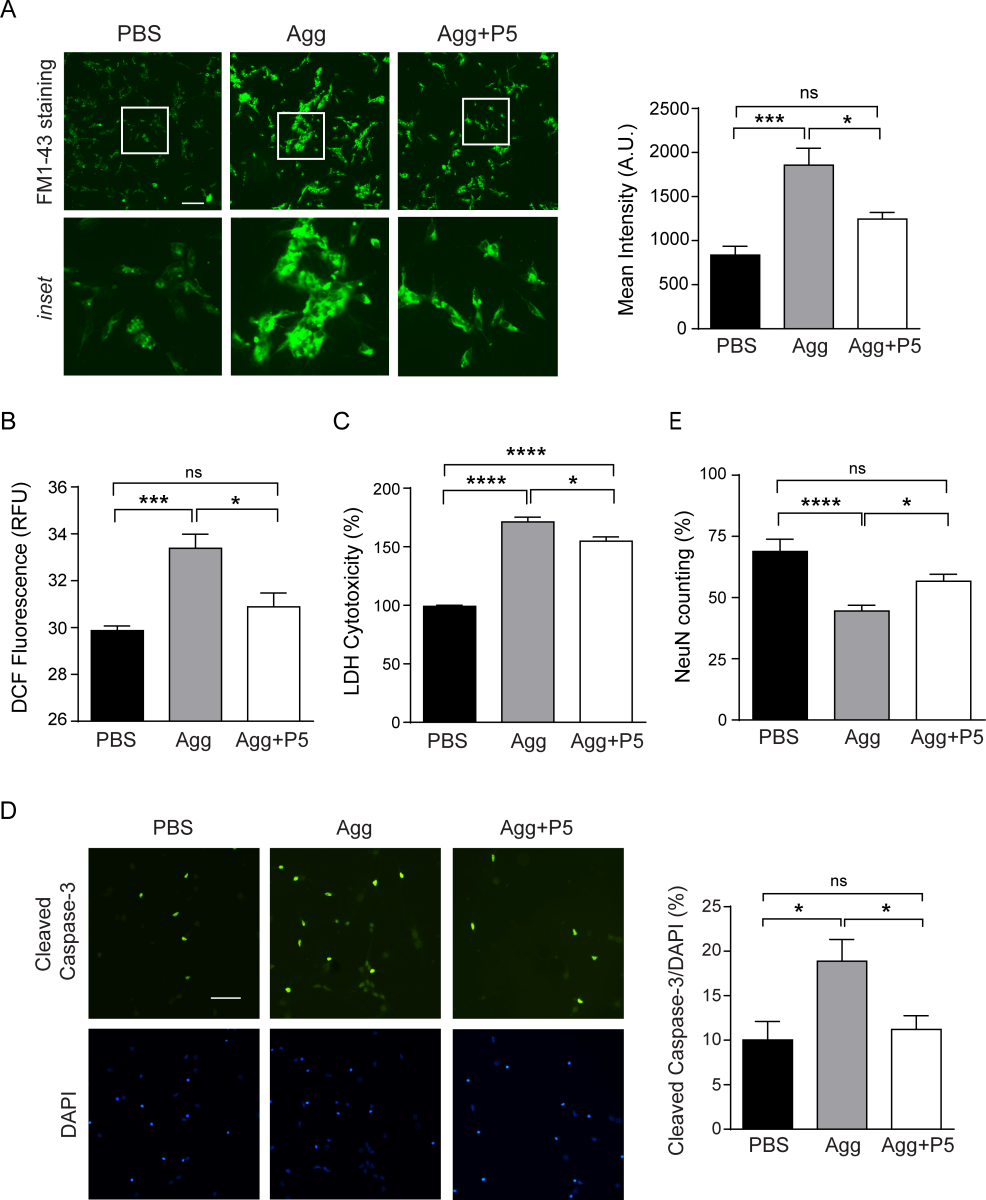
VAMP2-derived peptide reduces cytotoxicity induced by α-syn aggregates in SH-SY5Y cells and cultured primary neurons. Neuroblastoma SH-SY5Y cells were treated with α-syn aggregates pre-incubated with or without peptide P5. After 5 h treatment, (A) FM1-43 staining was performed (Scale bar, 50 µm) or (B) intracellular ROS levels were measured. (C) Pre-incubated α-syn aggregates with or without peptide P5 were treated cortical neuronal cells at DIV7 for 2 days consecutively and LDH cytotoxicity assay was performed. α-Syn aggregates toxicity was also evaluated by immunostaining of (D) cleaved caspase-3 and (E) NeuN in cortical neuronal cells at DIV7 after 3 days consecutive treatment. Scale bar, 50 µm. ****, p < 0.0001, ***, p < 0.001, *, p < 0.05, by ANOVA with Tukey's multiple comparisons test. Values indicate mean ± SEM.

To test whether α-syn aggregates interfere with the vesicular release in cells, we used FM1-43, a well-known tool to track exocytosis, endocytosis, and recycling of vesicle [50], in SH-SY5Y cells. As shown in Fig 7A, α-syn aggregates-treated cells retained more FM1-43 dye than control cells, indicating that the vesicular release was remarkably hampered by α-syn aggregates treatment. However, the retention of dye was reduced in cells treated with α-syn aggregates pre-incubated with peptide P5. This result implicated that peptide P5 prevented the impairment of vesicular release by α-syn aggregates. Next, intracellular reactive oxygen species (ROS) levels reflecting the cellular toxicities were measured in SH-SY5Y cells. After 5 h incubation, α-syn aggregates caused an elevation in ROS levels, as previously reported [36], but peptide P5 reduced intracellular ROS levels induced by α-syn aggregates (Fig 7B).

We further performed the functional assay in primary cultured neuronal cells. Lactate dehydrogenase (LDH) cytotoxicity was significantly high in response to α-syn aggregates, while peptide P5 reduced LDH release induced by α-syn aggregates after 2 days treatment (Fig 7C). A similar result was also observed in the immunostaining of cleaved caspase-3 as an apoptotic marker after 3 days treatment. As shown in Fig 7D, cleaved caspase-3-positive neurons were increased in α-syn aggregates-treated cells, whereas peptide P5-coated α-syn aggregates reduced the cleaved caspase-3 staining. Finally, assessment of cell survival using NeuN-positive counting showed a similar trend (Fig 7E). Altogether, these results supported that peptide P5 was efficient to suppress cellular toxicity mediated by α-syn aggregates.

## Discussion

In this study, we investigate a mechanism by which α-syn aggregates affect neurotoxicity through sequestration of functional cellular proteins such as VAMP2. Furthermore, we show that synthetic small peptide derived from VAMP2 is able to prevent neuronal damage mediated by α-syn aggregates.

A number of previous studies have reported that α-syn can adhere together under pathophysiological conditions, thereby producing a diverse form of toxic α-syn aggregates [1, 3, 28]. It has been also known that α-syn aggregates can interact with many cellular components directly to lead cellular dysfunction [33, 45]. In normal condition, C-terminal α-syn monomer binds to N-terminal VAMP2 for proper synaptic function [44], but short rod shape of α-syn aggregates inhibit the lipid mixing process of SNARE complex by interacting with the N-terminal domain of VAMP2 [33]. However, interestingly, our study shows that spherical shaped α-syn aggregates interact with VAMP2 via C-terminal domain of SNARE motif (Fig 6). Although VAMP2 bound to PFF, peptide P5 did not prevent the binding of VAMP2 to PFF which is known to interact through the N-terminal domain of VAMP2. This finding provides additional biochemical characteristics of α-syn aggregates interfering VAMP2 function. Another interesting point is that during amyloidogenic aggregate generation using artificial β sheet protein, globular aggregates are the most pronounced form with hydrophobic surfaces [51], further indicating that the spherical form of α-syn aggregates could have similar biochemical features.

Although it has been known that monomeric α-syn binds to VAMP2 [44], there was no direct binding in our assay condition that was designed for examining SDS-resistant binding (Fig 3). Our result was also supported by another recent study that VAMP2 preferentially binds to oligomeric form of α-syn rather than monomeric form [45]. Moreover, it is fairly comparable that α-syn oligomers prepared without dopamine with different morphology have the same binding effect on VAMP2 (S5 Fig). In addition, the strong binding between α-syn aggregates and VAMP2 under a broad range of temperature and pH (Fig 2) suggests that this type of aggregates can last in the *in vivo* condition without losing the sequestration capacity.

Previously, Volpicelli-Daley L *et al*. have shown that exogenous α-syn PFF is internalized by cultured neuronal cells and induce synaptic dysfunction and neuronal death [22]. They showed certain synaptic protein levels including SNAP25 and VAMP2 are selectively reduced in α-syn PFF-treated cells during the cytotoxic process with the explanation of the reduced neuronal excitability and connectivity [22]. In agreement with the study, we also observed the reduction of neurotransmitter release and significant cytotoxicity in the α-syn aggregate-treated neurons (Fig 4). These phenomena could be explained by a mechanism through which specific proteins such as SNAP25 and VAMP2 are selected as a target for the sequestration of α-syn aggregates as shown *in vitro* and *in vivo* (Figs 1, 4 and 5). In particular, it was meaningful for us to demonstrate that the aggregates generated *in vitro* exhibit similar characteristics to those formed *in vivo* PD mouse tissues.

Although extensive studies have been carried out on α-syn aggregates, little is known about how the aggregates acquire such specific target selectivity. As a target for the sequestration by α-syn aggregates, VAMP2 is one of the vulnerable synaptic proteins which easily undergoes proteolysis by neurotoxins like botulinum neurotoxin and tetanus neurotoxin known as inhibitors of synaptic vesicle release [52]. In particular, VAMP2 possesses specific cleavage sites for these neurotoxins within the cytoplasmic portion and these cleavage sites are mainly located at the end of the coiled-coil SNARE motif in VAMP2 [46]. Interestingly, the sequence of peptide P5 (^83^KLKRY^87^) used in our study is exactly corresponding to the cleavage site for antarease metalloprotease from Tityus serrulatus venom [46], indicating that this enzyme and α-syn aggregates may share similar feature to interact with VAMP2 in the pathology.

Considering such selective, but multiple binding partners to α-syn aggregates among SNARE complex components (Fig 1), it is fairly reasonable to speculate the presence of many other unknown target proteins in the cells. From the screening of cortical neuronal lysates, EF1α, which is important for protein synthesis and thus general cellular function, was identified as a major target for the sequestration by α-syn aggregates (data not shown). Recently, Shrivastava *et al*. showed that extracellular α-syn assemblies interact with plasma membrane protein α3-Na^+^/K^+^-ATPase leading to clustering and finally result in reduced pumping activity [53]. This is supportive of our study by emphasizing the sequestration effect of α-syn aggregates. Currently, it is unclear how many cellular proteins are responsive to the binding ability of α-syn aggregates and what biochemical properties such as common binding motif are involved in this selective sequestration. Even though Aβ aggregates failed to bind with VAMP2 in our study (Fig 3), numerous binding targets of Aβ aggregates were successfully screened and identified [51]. Therefore, additional studies are needed to determine the entire pool of target proteins to α-syn aggregates in the neuronal cells as well as other cell types in the brain. Since α-syn aggregates are known to play a pathological role through cell-to-cell transmission [22, 25], probably the aggregates may influence non-neuronal cells, suggesting that it will be of interest to characterize the novel binding candidates from those cells.

Recent studies have provided diverse therapeutic approaches to treat neurodegenerative diseases induced by abnormal protein aggregation. Small molecules have been reported to inhibit the formation of Aβ aggregates or remove pre-existing Aβ aggregates through disaggregation [54, 55]. To reduce tau expression in a mouse model of human tauopathy, antisense oligonucleotides have been developed as a useful tool for AD treatment [56]. Micro RNA-based gene therapy has been employed to reduce α-syn accumulation in dopaminergic neurons [57]. Additionally, immunotherapy has been demonstrated that α-syn antibodies block cell-to-cell transmission and thus prevent α-syn pathology [48, 58]. Interestingly, a natural product with anticancer and antiviral activity has been reported to inhibit the initiation of α-syn aggregation and suppress its toxicity by displacing α-syn from lipid membranes [59]. As a new therapeutic approach, we show that specific blocking peptide derived from VAMP2 inhibits in part the sequestration effect of α-syn aggregates in the cell-free system and neuronal cells, and alleviates cellular toxicity mediated by α-syn aggregates (Figs 6 and 7). Overall reduced toxicity seems to be due to the less cellular uptake of α-syn aggregates coated with hydrophilic peptide P5 (S8 Fig). Therefore, peptide P5 may play a preventive role in α-syn pathology by blocking cell-to-cell transmission.

Considering the eventual loss of dopaminergic neurons in the PD patient, prevention or at least delayed onset of cell death by lowering α-syn aggregates-mediated toxicity could be a useful strategy. Recently, Mor *et al*. have shown that dopamine-induced toxicity is dependent on α-syn, which suggests a synergistic toxic effect of dopamine and α-syn [60]. They have also shown that dopamine can modify α-syn oligomer conformation *in vivo*, which is thought to be neurotoxic species and a promising new target for PD treatment [60]. Consistent with this study, our findings provide biochemical and/or functional characteristics of dopamine-induced α-syn aggregates and the potential blocking peptide as a therapeutic approach. In this study, we focused on only synaptic proteins as α-syn aggregates target, however, probably many unknown targets in regulating critical cell survival would exist.

Although peptide P5 failed to recover the glutamate release in the damaged neurons by α-syn aggregates (S7 Fig), it could rescue vesicular release impaired by α-syn aggregates (Fig 7A). This might be explained by the possibility that key molecules involved in glutamate release may be aside from the beneficial role of peptide P5. Indeed, α-syn regulates neurotransmitter release by affecting many components such as synaptic vesicle clustering, the recycling pool size, and membrane lipid of organelles [21, 24, 35, 61]. Another potential possibility is that undefined toxic motifs of α-syn aggregates could not be blocked by peptide P5. To solve this issue, definitive information about the binding motif of α-syn aggregates from entire target pool will be useful in the future. In addition, as a useful therapeutic for PD, peptide P5 should trespass into the brain through the blood-brain barrier (BBB). Since peptide P5 displays relatively high hydrophilicity, it may not be easy to penetrate BBB. When we performed the intravenous injection of FITC-conjugated P5, we failed to observe FITC signal in the brain parenchyma (data not shown), indicating that peptide P5 alone may not be appropriate for crossing BBB. Probably this limitation could be solved by redesigning peptide P5 such as adding cell-penetrating peptide or increasing hydrophobicity.

Lastly, because biochemical features of α-syn aggregates are extensively diverse and variable, it is extremely difficult to find proper therapeutics. Through understanding unique characteristics of α-syn aggregates described in this study, we suggest that small peptide, as a tool to mimic and competitively interact with the target proteins of α-syn aggregates, is useful and could be a potential strategy for the development of new PD therapeutics.

## Supporting information

S1 Fig The purity of recombinant proteins. All recombinant proteins used in this study were prepared as described in the Materials and methods and showed high purity as shown in SDS-gel stained by SyproOrange.
(PDF)

S1 Table Antibodies used in this study. (DOCX)

S2 Fig Specific SNARE components bind to α-syn aggregates directly. (A) After binding assay with α-syn aggregates as shown in Fig 1D, immunoblot images of anti-αSyn and anti-VAMP2, respectively, were changed to colored images in ImageJ. Superimposed image (right) of α-syn (left) and VAMP2 (middle) showed direct binding of VAMP2-aggregates. (B) Recombinant SNAP25 was incubated with α-syn aggregates at 37°C for 2 h and then the samples were subjected to western blot analysis. To detect bound SNAP25, the membrane was cut and exposed longer. Notice that in the unboiled condition, SNAP25 oligomeric forms were also detected in 75 kDa and 150 kDa (left lane).
(PDF)

S3 Fig Dopamine-induced α-syn aggregates are highly pure and stable. (A) Profile of size-exclusion chromatography showed high yield of aggregates. (B) Western blot analysis using anti-αSyn antibody showed highly pure and stable aggregates.
(PDF)

S4 Fig The spherical form of α-syn aggregates are generated in the absence of dopamine. (A) Profile of size-exclusion chromatography showed that most proteins remained as monomeric form. (B) Low level of aggregated form (fraction # 10) was detected by western blot analysis after longer exposure of cut membrane. (C) The spherical morphology was confirmed by TEM analysis. (D) VAMP2 binding assay showed that VAMP2 bound to this aggregates with relatively low efficiency. The reduced level of free VAMP2 was observed (red arrow in left) and the bound VAMP2 to α-syn aggregates without dopamine was shown in the cut membrane by longer exposure (right).
(PDF)

S5 Fig α-Syn PFF with different morphology shows a similar effect on VAMP2 binding and cellular toxicity. (A) The morphology of α-syn PFF was examined by TEM. Scale bar, 100 nm. (B) VAMP2 bound to α-syn PFF as well as α-syn aggregates in a cell-free system (left). Dopamine-induced aggregates showed consistent size of the large oligomer, whereas PFF showed smear pattern of bands in diverse size (right). (C) Intracellular calcium levels were measured in neuronal cells after 15 days incubation of α-syn aggregates or α-syn PFF at the same concentrations (3 µg/ml) using Fluo-4 AM. Calcium influx was increased in response to α-syn aggregates and α-syn PFF. F_0_ and F_t_ represent fluorescence intensity of the indicator at 0 min and 60 min, respectively. **, p < 0.01 by ANOVA with Tukey's multiple comparisons test (n = 7). Values indicate mean ± SEM.
(PDF)

S6 Fig Peptide P5 blocks the sequestration of SNAP25 by α-syn aggregates in a cell-free system. α-Syn aggregates were pre-incubated with or without peptide P5 at RT for 1 h. Then, SNAP25 was added in the reaction and incubated at 37°C for 2 h. Bound SNAP25 was detected from the longer exposure of cut membrane. Quantitative results were also shown in the graph (right). ****, p < 0.0001, **, p < 0.01, by ANOVA with Tukey’s multiple comparisons test (n = 3).
(PDF)

S7 Fig Peptide P5 is not effective in glutamate assay but protects cells from cytotoxicity. (A) Pre-incubated α-syn aggregates with or without peptide P5 (1 mM) were treated neuronal cells at DIV7 for 3 days consecutively and glutamate levels were determined. Peptide P5 did not show significant recovery of impaired glutamate levels mediated by α-syn aggregates. ****, p < 0.0001, ***, p < 0.001, by ANOVA with Tukey’s multiple comparisons test (n = 4). (B) Neurons were delivered pre-incubated α-syn aggregates with or without peptide P5 using protein transfection reagent and cell morphology was assessed by phase contrast microscopy after 3 h. Neuronal cells transfected peptide P5 alone (left) showed normal morphology, while high-dose transfection of α-syn aggregates (middle) resulted in rapid cell death. However, cells transfected α-syn aggregates with P5 (right) were protected from cell death. Scale bar, 100 µm.
(PDF)

S8 Fig Peptide P5 partially inhibits the internalization of α-syn aggregates in cortical neurons. α-Syn aggregates with (lanes 4, 5) or without (lane 3) peptide P5 were treated to cortical neuronal cells. Before adding α-syn aggregates, peptide P5 was pre-treated on neurons for 30 min (lane 4, *) or pre-incubated with α-syn aggregates for 1 h, then treated on neurons (lane 5, **). After 3 h incubation, cells were lysed and western blot analysis was performed. Both pre-treatment and pre-incubation of peptide P5 conditions, α-syn aggregates level was less detected.
(PDF)

S1 File NC3Rs ARRIVE guidelines checklist. (PDF)
