# Supplementary figures and images for: Sequestration of synaptic proteins by alpha-synuclein aggregates leading to neurotoxicity is inhibited by small peptide
Source: PLoS One. 2018 Apr 2;13(4):e0195339. doi: 10.1371/journal.pone.0195339 (PMC5880409; doi:10.1371/journal.pone.0195339)

S1 Fig

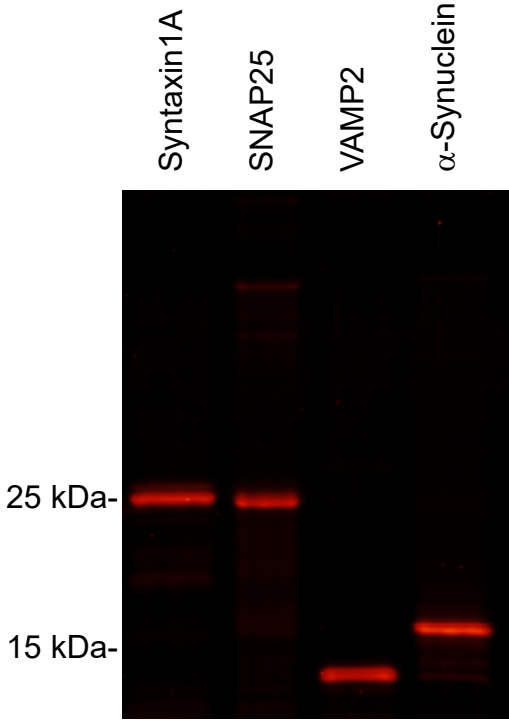

Supplement: S1 Fig — All recombinant proteins used in this study were prepared as described in the Materials and methods and showed high purity as shown in SDS-gel stained by SyproOrange. (PDF) [file pone.0195339.s001.pdf]

S2 Fig

A

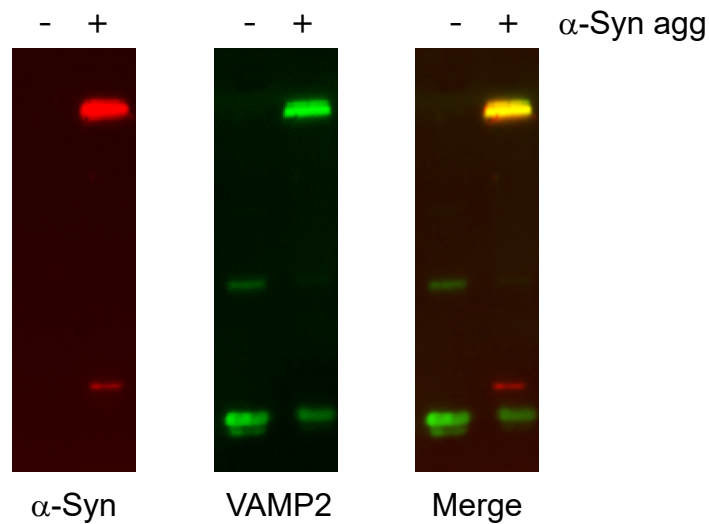

B

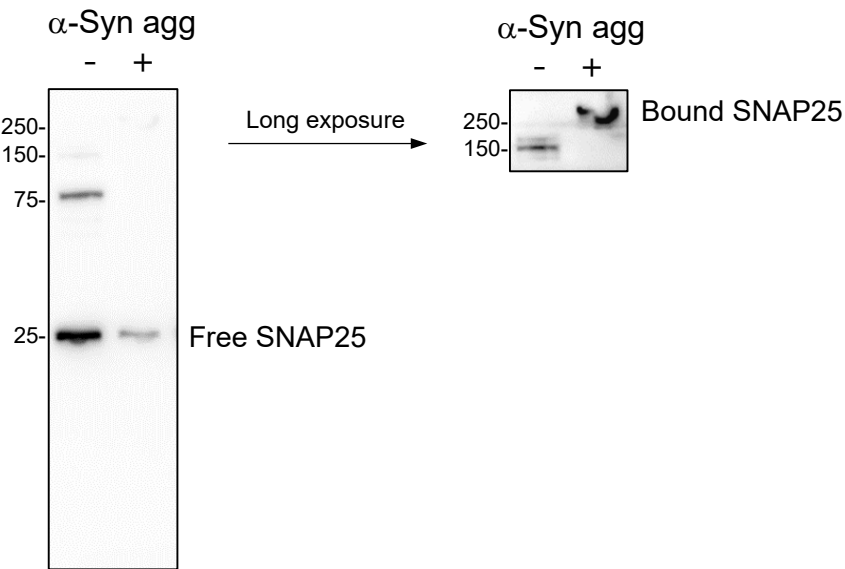

Supplement: S2 Fig — (A) After binding assay with α-syn aggregates as shown in Fig 1D, immunoblot images of anti-αSyn and anti-VAMP2, respectively, were changed to colored images in ImageJ. Superimposed image (right) of α-syn (left) and VAMP2 (middle) showed direct binding of VAMP2-aggregates. (B) Recombinant SNAP25 was incubated with α-syn aggregates at 37°C for 2 h and then the samples were subjected to western blot analysis. To detect bound SNAP25, the membrane was cut and exposed longer. Notice that in the unboiled condition, SNAP25 oligomeric forms were also detected in 75 kDa and 150 kDa (left lane). (PDF) [file pone.0195339.s003.pdf]

S3 Fig

A

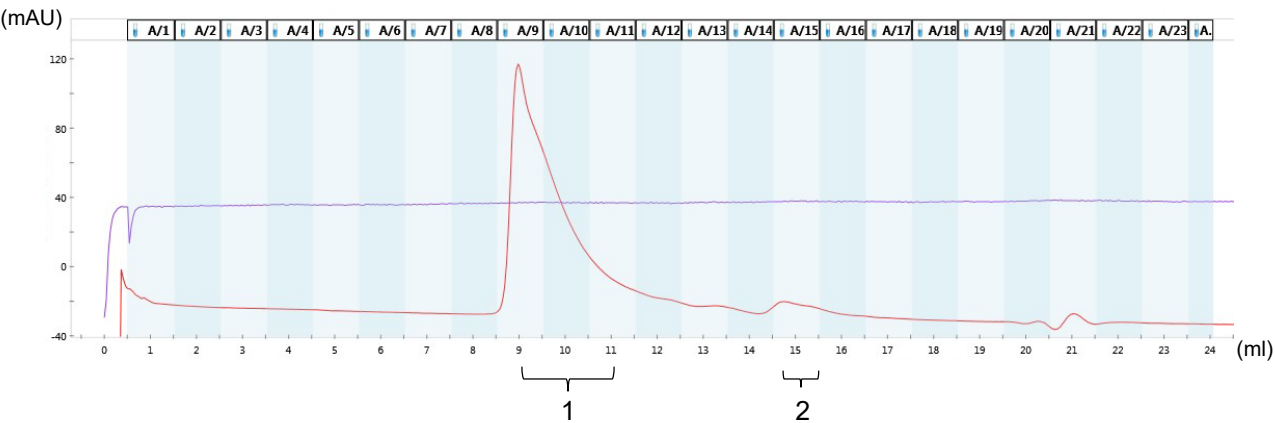

B

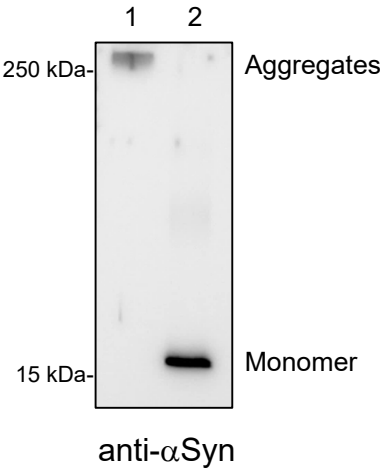

Supplement: S3 Fig — (A) Profile of size-exclusion chromatography showed high yield of aggregates. (B) Western blot analysis using anti-αSyn antibody showed highly pure and stable aggregates. (PDF) [file pone.0195339.s004.pdf]

S4 Fig

A

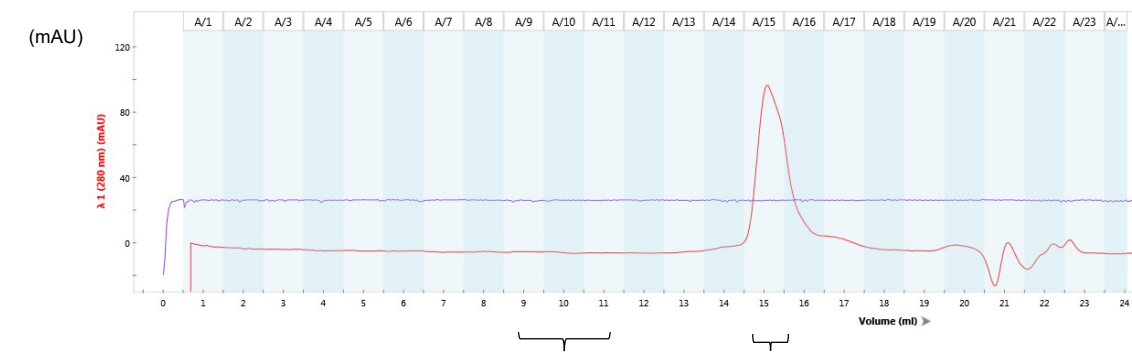

B

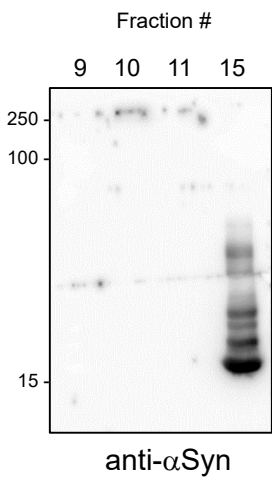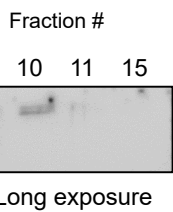

C

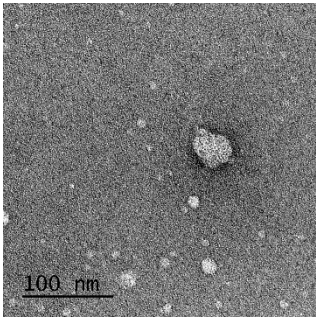

D

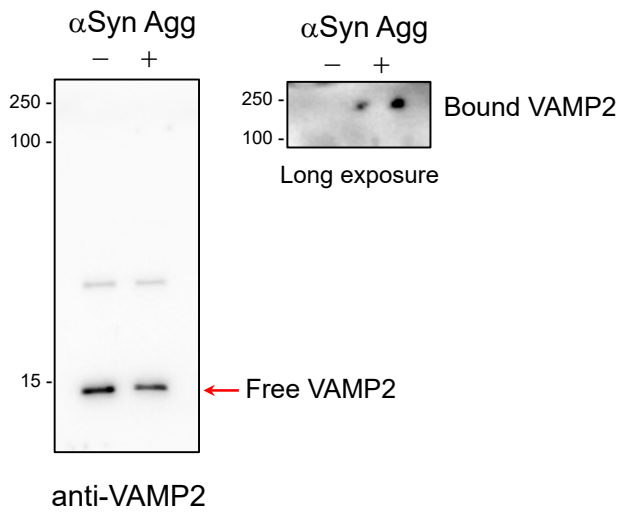

Supplement: S4 Fig — (A) Profile of size-exclusion chromatography showed that most proteins remained as monomeric form. (B) Low level of aggregated form (fraction # 10) was detected by western blot analysis after longer exposure of cut membrane. (C) The spherical morphology was confirmed by TEM analysis. (D) VAMP2 binding assay showed that VAMP2 bound to this aggregates with relatively low efficiency. The reduced level of free VAMP2 was observed (red arrow in left) and the bound VAMP2 to α-syn aggregates without dopamine was shown in the cut membrane by longer exposure (right). (PDF) [file pone.0195339.s005.pdf]

S5 Fig

A

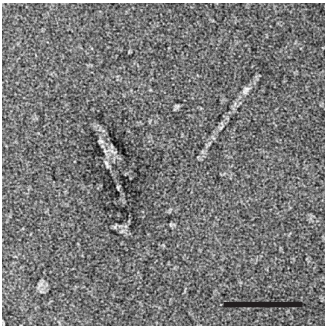

B

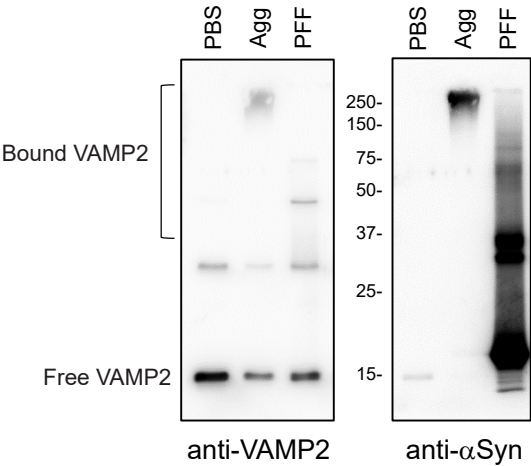

C

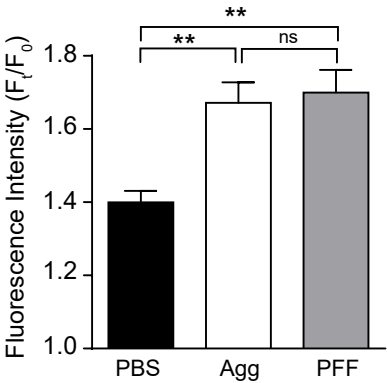

Supplement: S5 Fig — (A) The morphology of α-syn PFF was examined by TEM. Scale bar, 100 nm. (B) VAMP2 bound to α-syn PFF as well as α-syn aggregates in a cell-free system (left). Dopamine-induced aggregates showed consistent size of the large oligomer, whereas PFF showed smear pattern of bands in diverse size (right). (C) Intracellular calcium levels were measured in neuronal cells after 15 days incubation of α-syn aggregates or α-syn PFF at the same concentrations (3 µg/ml) using Fluo-4 AM. Calcium influx was increased in response to α-syn aggregates and α-syn PFF. F0 and Ft represent fluorescence intensity of the indicator at 0 min and 60 min, respectively. **, p < 0.01 by ANOVA with Tukey's multiple comparisons test (n = 7). Values indicate mean ± SEM. (PDF) [file pone.0195339.s006.pdf]

S6 Fig

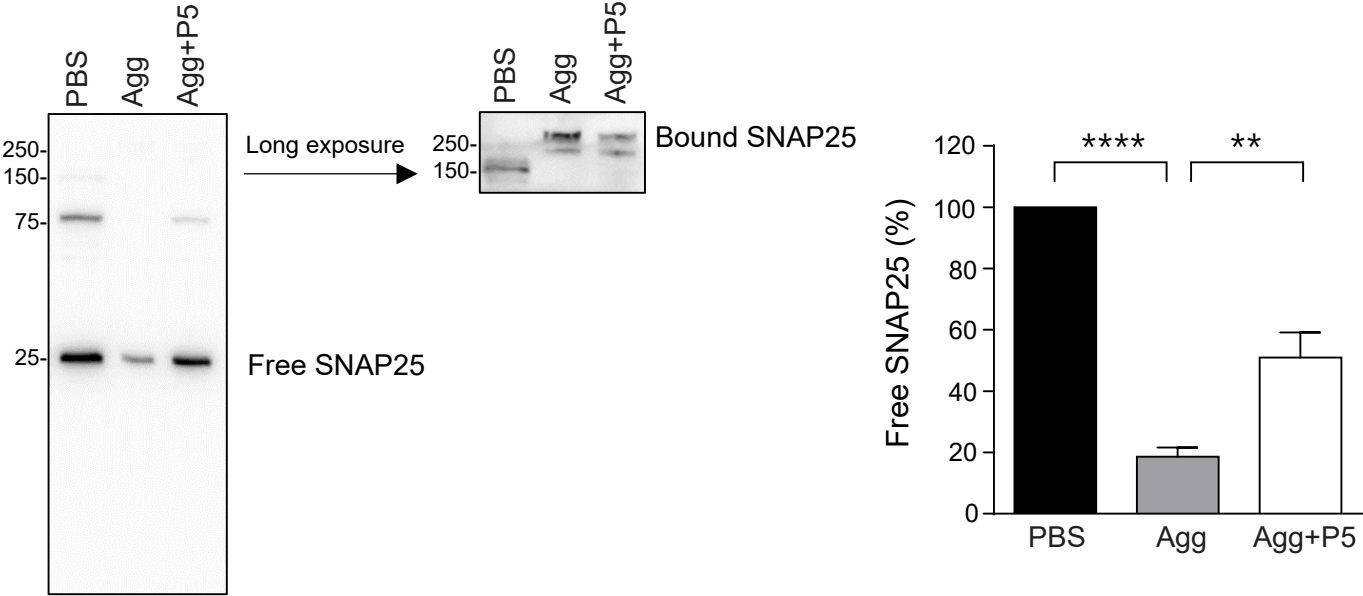

Supplement: S6 Fig — α-Syn aggregates were pre-incubated with or without peptide P5 at RT for 1 h. Then, SNAP25 was added in the reaction and incubated at 37°C for 2 h. Bound SNAP25 was detected from the longer exposure of cut membrane. Quantitative results were also shown in the graph (right). ****, p < 0.0001, **, p < 0.01, by ANOVA with Tukey’s multiple comparisons test (n = 3). (PDF) [file pone.0195339.s007.pdf]

S7 Fig

A

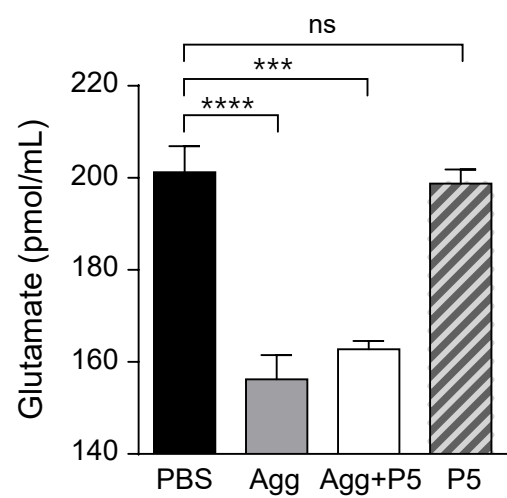

B

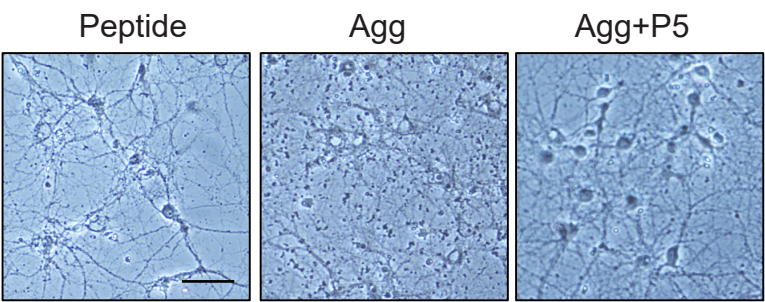

Supplement: S7 Fig — (A) Pre-incubated α-syn aggregates with or without peptide P5 (1 mM) were treated neuronal cells at DIV7 for 3 days consecutively and glutamate levels were determined. Peptide P5 did not show significant recovery of impaired glutamate levels mediated by α-syn aggregates. ****, p < 0.0001, ***, p < 0.001, by ANOVA with Tukey’s multiple comparisons test (n = 4). (B) Neurons were delivered pre-incubated α-syn aggregates with or without peptide P5 using protein transfection reagent and cell morphology was assessed by phase contrast microscopy after 3 h. Neuronal cells transfected peptide P5 alone (left) showed normal morphology, while high-dose transfection of α-syn aggregates (middle) resulted in rapid cell death. However, cells transfected α-syn aggregates with P5 (right) were protected from cell death. Scale bar, 100 µm. (PDF) [file pone.0195339.s008.pdf]
