# Supplementary material for: Sequestration of synaptic proteins by alpha-synuclein aggregates leading to neurotoxicity is inhibited by small peptide
Source: PLoS One. 2018 Apr 2;13(4):e0195339. doi: 10.1371/journal.pone.0195339 (PMC5880409; doi:10.1371/journal.pone.0195339)
Supplement: S1 Table — (DOCX) [file pone.0195339.s002.docx]

**S1 Table. Antibodies used in this study.**

| Antibodies | Source (Cat. No.) | Host | Dilution |
| --- | --- | --- | --- |
| α-Syn | Santa Cruz Biotechnology (sc-7011-R)  BD Biosciences (610787)  BioLegend (824301) | Rabbit  Mouse  Mouse | 1:1000 (WB)  1:1000 (WB)  1:500 (IHC) |
| P-α-Syn (Ser129) | Abcam (ab168381) (Ref. 49) | Rabbit | 1:500 (ICC) |
| VAMP2 | Abcam (ab181869)  Abcam (ab181754) | Rabbit  Mouse | 1:5000 (WB), 1:150 (IP), 1:500 (IHC)  10 µg/ml (ICC) |
| SNAP25 | Santa Cruz Biotechnology (sc-376713) | Mouse | 1:1000 (WB) |
| Syntaxin1A | Cell Signaling (13002S) | Rabbit | 1:1000 (WB) |
| Aβ | BioLegend (SIG-39320) | Mouse | 1:1000 (WB) |
| Cleaved Caspase-3 | Cell Signaling (9661) | Rabbit | 1:500 (ICC) |
| His-Tag | Thermo Fisher (MA1-21315) | Mouse | 1:1000 (WB) |
