# Supplementary material for: Sequestration of synaptic proteins by alpha-synuclein aggregates leading to neurotoxicity is inhibited by small peptide
Source: PLoS One. 2018 Apr 2;13(4):e0195339. doi: 10.1371/journal.pone.0195339 (PMC5880409; doi:10.1371/journal.pone.0195339)
Supplement: S8 Fig — α-Syn aggregates with (lanes 4, 5) or without (lane 3) peptide P5 were treated to cortical neuronal cells. Before adding α-syn aggregates, peptide P5 was pre-treated on neurons for 30 min (lane 4, *) or pre-incubated with α-syn aggregates for 1 h, then treated on neurons (lane 5, **). After 3 h incubation, cells were lysed and western blot analysis was performed. Both pre-treatment and pre-incubation of peptide P5 conditions, α-syn aggregates level was less detected. (PDF) [file pone.0195339.s009.pdf]

S8 Fig

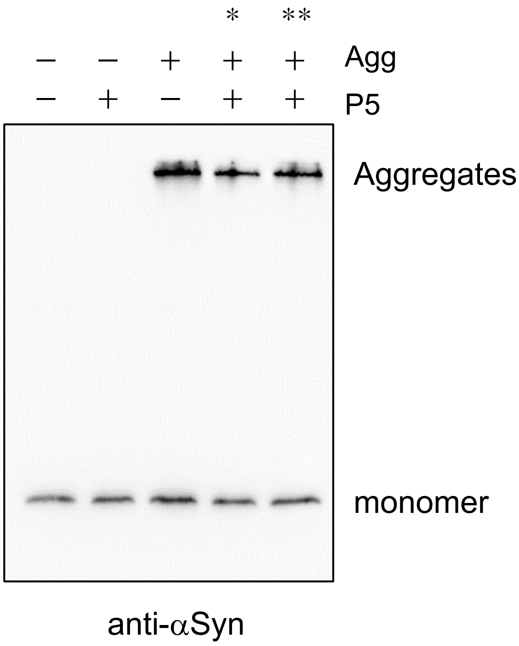

* ; Pre-treatment of P5 to the cell before Agg addition

** ; Pre-incubation of P5 with Agg before treatment to the cell
